# PINK1-PARK2-mediated mitophagy in COPD and IPF pathogeneses

**DOI:** 10.1186/s41232-018-0077-6

**Published:** 2018-10-24

**Authors:** Kazuya Tsubouchi, Jun Araya, Kazuyoshi Kuwano

**Affiliations:** 1 Division of Respiratory Diseases, Department of Internal Medicine, Jikei University School of Medicine, 3-25-8 Nishi-shimbashi, Minato-ku, Tokyo, 105-8461 Japan; 2 Research Institute for Diseases of the Chest, Graduate School of Medical Sciences, Kyushu University, Fukuoka, Japan

**Keywords:** Mitophagy, PARK2, PINK1, COPD, IPF

## Abstract

Mitochondria regulate not only cell functions through energy generation but also aging-associated cell phenotypes. Impaired mitochondrial structural and functional integrity accompanied by excessive mitochondrial reactive oxygen species (mtROS) production is associated with enhanced programmed cell death (PCD) and cellular senescence. Dysregulation of mechanisms for mitochondrial integrity, including mitophagy, induces accumulation of mitochondrial damage. Mitophagy is a highly conserved mechanism of selectively delivering damaged mitochondria for lysosomal degradation and is mainly governed by phosphatase and tensin homolog (PTEN)-induced putative protein kinase 1 (PINK1) and PARK2. Accumulating evidence suggests that PINK1-PARK2-mediated mitophagy has an important role in the pathogenesis of aging-associated pulmonary disorders, represented by chronic obstructive pulmonary disease (COPD) and idiopathic pulmonary fibrosis (IPF).

COPD characterized by progressive airflow limitation is mainly caused by cigarette smoke (CS) exposure, and accumulation of damaged mitochondria in bronchial epithelial cells (BEC) has been demonstrated. Intriguingly, both enhanced and impaired mitophagy have been implicated in COPD pathogenesis. Enhanced mitophagy induced by increased PINK1 expression has been associated with programmed necrosis, necroptosis. On the other hand, reduced PARK2 levels were linked to insufficient mitophagy, resulting in accelerated cellular senescence in BEC. Although dominant involvement of PCD and cellular senescence remains unclear, PINK1-PARK2-mediated mitophagy regulates mitochondrial ROS and cell fate during COPD pathogenesis.

Involvement of insufficient mitophagy has been proposed in lung fibrosis development during IPF pathogenesis. Accumulation of dysmorphic mitochondria and increased ROS production linked to decrease in PINK1 expression were demonstrated in type II alveolar epithelial cells (AECIIs) in IPF lungs, which can be associated with enhanced apoptosis and cellular senescence. Furthermore, reduced PARK2 expression levels have been shown in myofibroblasts in IPF lungs. Insufficient mitophagy caused by PARK2 deficiency induced mtROS production with concomitantly activated platelet-derived growth factor receptor (PDGFR)/mammalian target of rapamycin (mTOR) signaling, resulting in increased myofibroblast differentiation and proliferation.

Inappropriate PINK1-PARK2-mediated mitophagy appears to be mainly responsible for regulating cell fate, including PCD, cellular senescence, and myofibroblast differentiation during COPD and IPF pathogeneses. Modalities to achieve specific and appropriate levels of PINK1-PARK2-mediated mitophagy activation may be a promising therapeutic option to regulate the aging-associated pathology, COPD, and IPF.

## Background

Mitochondria regulate cellular bioenergetics through the process of oxidative phosphorylation (OXOHOS), in which ATP is formed as a result of the transfer of electrons [1]. However, accumulating evidence suggests the pivotal role of mitochondria in regulating not only cell functions through energy generation but also aging-associated cell phenotypes including cellular senescence [2]. Although mitochondria are the main sources of endogenous reactive oxygen species (ROS) during electron transport, mitochondrial DNA (mtDNA) has been known to be more susceptible to oxidative injury, which is attributed to lack of protective histones and paucity of repair mechanisms, indicating the existence of vicious cycle of mitochondrial damage and ROS production. Accumulation of mitochondrial damage is associated with excessive ROS production, resulting in oxidative damage to cellular components, including DNA, lipids, and proteins [2]. Mitochondrial ROS can also lead to the generation of single-strand breaks in telomere regions, resulting in telomere shortening, suggesting the causal association between mitochondrial damage and telomere health [3]. Excessive mitochondrial damage has also been widely implicated in programmed cell death (PCD). Accordingly, mitochondrial damage should be adequately regulated for preventing accelerated cellular senescence and PCD.

Mitochondrial integrity is orchestrated by complex regulatory mechanisms, including biogenesis, dynamics of fusion and fission, and degradation. Dysregulation of these regulatory mechanisms has been widely implicated in aging-associated disease pathogeneses, including pulmonary disorders [1]. Mitochondria selective autophagy-lysosomal degradation, known as mitophagy, plays a crucial role in degradation of damaged mitochondria and is mainly governed by posttranslational modifications of phosphatase and tensin homolog (PTEN)-induced putative protein kinase 1 (PINK1) and Parkinson disease 2 (PARK2) [4–8].

In this review, we focus on dysregulation of mechanisms for mitochondrial integrity, including PINK1-PARK2-dependent mitophagy in association with progression of cellular senescence, cell death, and myofibroblast differentiation as a part of pathogenesis of aging-associated pulmonary disorders, represented by chronic obstructive pulmonary disease (COPD) and idiopathic pulmonary fibrosis (IPF).

## Accumulation of mitochondrial damage in COPD and IPF

Several studies including ours showed involvement of cellular senescence in the pathogeneses of aging-associated pulmonary disorders of COPD and IPF [9–15]. Cellular senescence is characterized by durable cell-cycle arrest and senescence-associated secretory phenotype (SASP) of excessive cytokine production [16]. Among a variety of proposed mechanisms, mitochondrial free radical theory (MFRTA) has been recognized to be a crucial mechanism for cellular senescence [2]. ROS are natural byproduct during OXOHOS, which have physiological roles in regulating cell signalings. In general, mitochondrial ROS (mtROS) can be increased by accumulation of damaged mitochondria, resulting in oxidative damage to DNA, lipids, and proteins in cell compartments [2]. Antioxidant MitoQ or acetyl-l-carnitine, which targeted damaged mitochondria with scavenging mtROS, delays both replicative senescence and stress-induced premature senescence [17–19]. Morphological alterations of damaged mitochondria, represented by enlargement, loss of cristae, and destruction of inner membrane, are demonstrated during aging [20]. Similar morphological alterations of mitochondria with increased mtROS have been detected in both COPD and IPF lung epithelial cells [21–23]. Aging is linked to not only increased mtROS but also reduced ATP production, which can be attributed to reduced antioxidant defense system and reduced electron transport chain (ETC) complex activity. Reduced ETC complex I and V activity in type II alveolar epithelial cells and reduced ATP content and oxygen consumption rate in fibroblasts are demonstrated in IPF lungs [1].

Due to lack of protective histones and paucity of repair mechanisms, mitochondrial DNA has been known to be more susceptible to oxidative injury. Accumulation of mutations and the deletion of mtDNA have been implicated in accelerated aging. mtDNA mutator mice without proofreading property of the mtDNA polymerase gamma (POLG) showed premature aging accompanied by high levels of point mutations [24], suggesting the causal link between accumulation of mtDNA damage and aging phenotypes. Actually, increased quantity of mtDNA with oxidative damage during aging and smoking stress has been demonstrated [25]. Substantial increase in mtDNA strand breaks and/or abasic sites was detected in lung tissues from COPD patients [26]. The authors speculated that genome and sequence-specific oxidative DNA damage could contribute to transcriptional dysregulation and cell fate decisions in COPD [26]. Mitochondrial SIRT3, a member of sirtuin family has been implicated in the regulation of lung fibrosis development with respect to regulating mtDNA damage via modulating 8-oxoguanine-DNA glycosulase-1 (OGG1) acetylation, a known DNA repair enzyme [20, 27]. SIRT3 deficient mice showed enhanced lung fibrosis development by bleomycin and asbestos exposure [20, 28]. Pharmacologic activation of SIRT3 mitigates organ fibrosis development [29]. Reduced SIRT3 expression accompanied by higher proportion of damaged mtDNA has been demonstrated in aging lungs, suggesting the potential causal link between reduced SIRT3-mediated accumulation of mtDNA damage and IPF development.

mtDNA contains CpG-rich sequences of bacterial molecular motifs, which may act as damage-associated molecular patterns (DAMPs) for innate immune response. In the setting of mtDNA spreading out of mitochondrial compartment because of mitochondrial damage, both cytoplasmic and circulating mtDNA may cause inflammatory responses to injury [30, 31]. Cigarette smoke (CS)-induced necroptosis in airway epithelial cells initiates inflammatory responses of cytokine production though releasing DAMPs, including mtDNA as a COPD pathogenesis [32]. Intriguingly, recent paper showed release of mtDNA from IPF fibroblasts and circulating mtDNA can be a biomarker for IPF severity, indicating potential association between circulating mtDNA of mitochondrial damage and innate immune response-mediated lung fibrogenesis [33].

## Mitochondrial biogenesis in COPD and IPF

Mitochondrial functional and morphological integrity is orchestrated by complex regulatory mechanisms, including biogenesis, dynamics of fusion and fission, and degradation. Mitochondria biogenesis for extending cellular energy production is governed by master regulators PPARγ coactivator-1α (PGC-1α) and PGC-1β in association with nuclear respiration factors (NRF) expression [1]. PGC-1 coactivator docking to specific transcription factors provides a platform for the recruitment of regulatory protein complexes that exert powerful effects on gene transcription for mitochondria biogenesis. PGC-1α and PGC-1β are preferentially expressed in tissues with high oxidative capacity, such as heart, slow-twitch skeletal muscle, and brown adipose tissue, where they serve critical roles in the regulation of mitochondrial functional capacity and cellular energy metabolism [34]. In general, the capacity for mitochondrial biogenesis declines during aging through the reductions of PGC-1α and PGC-1β. Aging-related decline in mitochondrial biogenesis linked to reduced PGC-1α has been implicated in IPF pathogenesis [1, 35]. Reduced PGC-1α expression levels have been demonstrated in both IPF lungs and bleomycin-induced lung fibrosis models. Furthermore, PGC-1α-deficient mice showed susceptibility to bleomycin-induced lung fibrosis [1, 35]. Interestingly, recent paper showed that thyroid hormone treatment attenuates bleomycin-induced lung fibrosis development via reducing alveolar epithelial cell apoptosis, which can be at least partly attributed to PGC-1α-mediated mitochondrial biogenesis accompanied by normalized mitochondrial morphological integrity [36]. Other mechanisms for attenuation of mitochondrial biogenesis has also reported in IPF pathogenesis, including NADPH oxidase-4 (NOX4)-mediated NRF2 and mitochondrial transcription factor A(TFAM) inhibition [1]. Accordingly, it is likely that impaired mitochondrial biogenesis has an essential role in regulating mitochondrial integrity with respect to IPF pathogenesis, and upregulation of PGC-1α for enhancing mitochondrial biogenesis can be a promising approach for IPF treatment.

On the other hand, it has been reported that mTOR-driven PGC-1β-dependent mitochondrial biogenesis may also have an essential role in cellular senescence progression induced by a variety of triggers [37]. Accumulation of damaged mitochondria has also been reported in COPD lungs, especially in association with accelerated cellular senescence [21]. Our experimental results using human bronchial epithelial cells (HBEC) demonstrated that accumulation of damaged mitochondria by CS exposure is responsible for cellular senescence progression, and PGC-1β-mediated mitochondrial biogenesis is involved in this process (paper in submission). Although precise mechanisms and roles remain elusive, it is plausible that PGC-1-mediated mitochondrial biogenesis can be beneficial for preventing apoptosis in IPF but may be harmful for accelerating cellular senescence in COPD pathogenesis.

## Mitochondrial dynamics in COPD and IPF

Mitochondrial morphological changes by dynamics of fusion and fission have been recognized to be a major mitochondrial quality control mechanism [38]. Mitochondrial elongation by fusion is an adaptive stress-resolving mechanism by exchanging the damaged mtDNA, proteins, and lipids between damaged and healthy mitochondria by mild oxidative stress. Elongated mitochondria are spared from autophagic degradation and have increased levels of ATP synthesis. Mitochondrial fragmentation by fission is induced in the setting of severe oxidative stress, which is associated with programmed cell death or elimination of damaged mitochondria via mitophagy [39]. Alterations in mitochondrial morphology have been implicated in ROS production in terms of COPD pathology [39]. Cigarette smoke can induce both mitochondrial fragmentation and elongation, which can be dependent on the level of oxidative stress [21, 22]. Mitochondrial dynamics is regulated by the balance between fusion-promoting proteins, including optic atrophy 1 (OPA1) and mitofusin (MFN), and fission-promoting proteins, including dynamin-related protein 1 (DRP1) and mitochondrial fission 1 protein (FIS1) [40]. CS-induced MFN/OPA1 expression causes hyperfusion of mitochondria accompanied by impaired stress resistance and cellular senescence in lung epithelial cells [22]. CS also induces mitochondrial fission by translocation of DRP1 to mitochondria, resulting in accumulation of fragmented mitochondria with increased ROS production and accelerated cellular senescence [21]. Furthermore, prolonged CS exposure induces FIS1 expression and reduction of MFN, resulting in mitochondrial fragmentation with enhanced ROS [39, 41]. Accordingly, mitochondrial morphological alterations can be involved in CSE-induced cellular senescence by ROS production during COPD pathogenesis; the level of oxidative stress can determine the fusion and fission status.

Mitochondrial morphological changes have also been reported in IPF lungs. Higher frequency of enlarged mitochondria and fusion tendency with increased mitochondrial area have been demonstrated in AECIIs in IPF lungs [23]. Those alterations may reflect accumulation of damaged and dysfunctional mitochondria conferred by insufficient mitophagic elimination, resulting in AECIIs apoptosis as a part of IPF pathogenesis [23]. Although metabolic shift from OXOHOS to glycolysis of less efficient ATP production has been reported during myofibroblast differentiation [42], mitochondrial morphological alterations remain unclear in IPF lung fibroblasts. Our in vitro experiments using lung fibroblasts, insufficient autophagy, and mitophagy induce mitochondrial elongation without apparent mitochondrial damage accompanied by increased myofibroblast differentiation and proliferation [40]. Thus, mitochondrial dynamics may contribute to cell phenotype regulation via various mechanisms in stimulus and cell type-specific manner, but precise role of mitochondrial dynamics in IPF pathogenesis remains unclear.

## PINK1-PARK2-mediated mitophagy

**Fig. 1:**
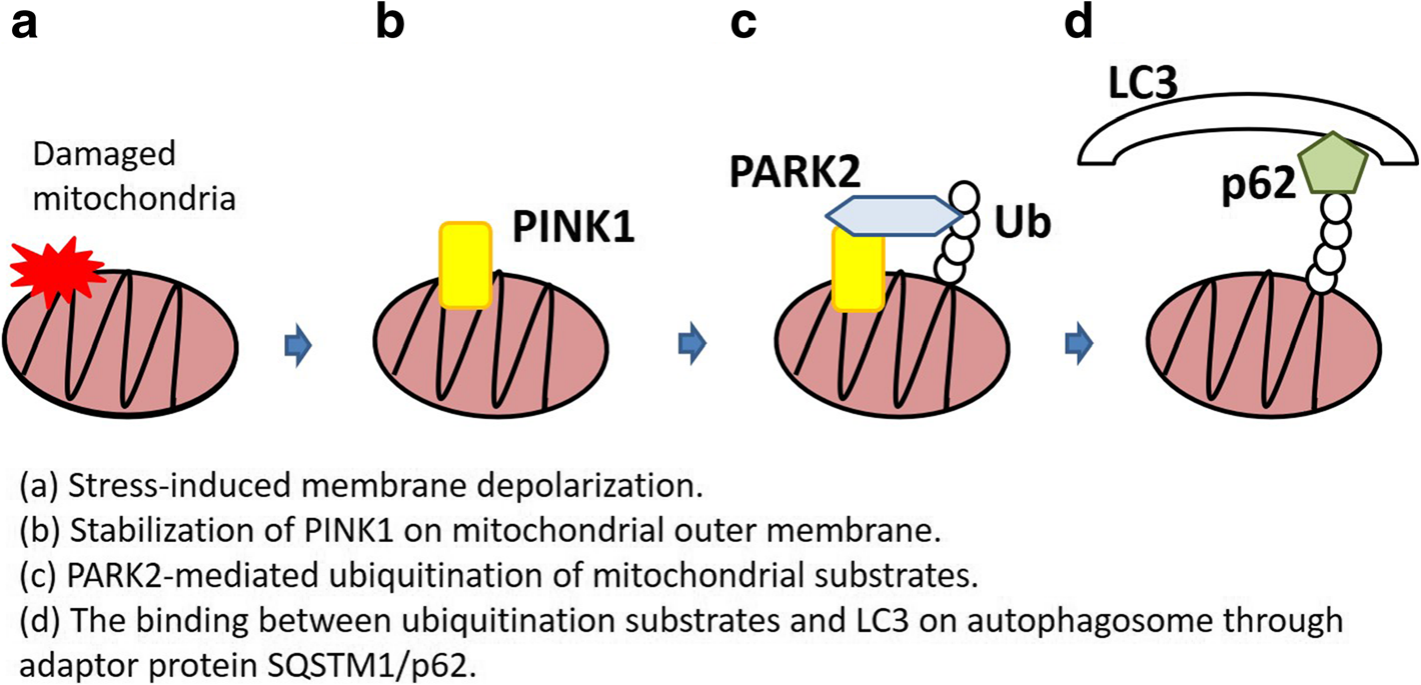
Mechanism of PINK1-PARK2-mediated mitophagy

Appropriate elimination of damaged and dysfunctional mitochondria plays a crucial role in preventing the release of proapoptotic proteins, mtROS and mtDNA, which can be causally linked to apoptosis, inflammasome activation, and cellular senescence [43–45]. Autophagy is a process of lysosomal self-degradation that helps maintain homeostatic balance between the synthesis, degradation, and recycling of cellular proteins and organelles [46]. Engulfment of cytoplasmic components by the isolation membrane (phagophore) is the initial step in autophagy and is followed by elongation and fusion, which results in the formation of double-membranous vesicles (autophagosome). Subsequent fusion of the autophagosome with the lysosome to form the autolysosome is essential for proper degradation [47]. Damaged mitochondria are mainly degraded via the mitochondria selective autophagy machinery known as mitophagy [4]. Mitophagy is a highly conserved mechanism of selectively delivering unwanted mitochondria for lysosomal degradation. Although PINK1-PARK2 pathway has an essential role in conducting mitophagy for removal of damaged mitochondria, there are several mechanisms for mitophagy in specific conditions. For example, BNIP3L, BNIP3, and the fun14 domain containing 1 (FUNDC1) have been demonstrated to be specific receptors for mitophagic recognition during red blood cell maturation, metabolic stress, and hypoxia. Furthermore, a recent paper showed the existence of PINK1-mediated PARK2-independent mitophagy via recruitment of autophagy receptors, Optineurin, including calcium binding and coiled-coil domain 2 (CALCOCO2/NDP52) and Optineurin (OPTN) [48–51]. Gene mutations of both PINK1 and PARK2 are coupled with Parkinson’s disease resulting from accumulation of damaged mitochondria, which can be attributed to insufficient mitophagic degradation [52]. Stress-induced membrane depolarization stabilizes PINK1 on mitochondrial outer membrane, resulting in recruitment of PARK2, an E3-ubiquitin ligase, to mitochondria. PARK2-mediated ubiquitination of mitochondrial substrates, including BCL2, mitofusins (MFN), and voltage-dependent anion channel (VDAC), is prerequisite for the binding of the adaptor protein SQSTM1/p62, which can recognize both ubiquitinated substrates and microtubule-associated protein 1 light chain 3 (MAP1LC3, LC3) on autophagosome [5, 8] (Fig. 1). In addition, PINK1-induced phosphorylated PARK2 (Ser65) triggers the degradation of mitochondrial fusion-promoting proteins, including MFN1 and MFN2, resulting in mitochondrial fission, which is prerequisite for conducting mitophagy [53]. Although the potential involvement of PINK1-PARK2-independent mitophagy remains to be elucidated, PINK1-PARK2 pathway-mediated mitophagy has been widely implicated in COPD and IPF pathogenesis through regulating cell fate, including programmed cell death, cellular senescence, and myofibroblast differentiation [14, 54].

## PINK1-PARK2-mediated mitophagy in COPD

COPD characterized by progressive airflow limitation is mainly caused by the noxious effects of CS exposure with rising incidence worldwide [15, 55]. CS, the major cause of COPD, is rich in toxic components, including ROS, and a variety of biological responses to cigarette smoke exposure have been demonstrated and oxidant-antioxidant imbalance has been widely implicated in COPD pathogenesis. Increased burden of oxidants in CS and ROS released from leukocytes and macrophages during inflammatory response have been considered as the source of ROS. However, oxidative stress can persist even after CS cessation, which can be attributed to endogenous ROS production, indicating the essential role of mtROS in COPD progression [56]. Chronic CS exposure can cause mitochondrial dysfunction in lung epithelial cells [22]. Intriguingly, it has been reported that lipophilic fraction in CS extract is directly responsible for a decrease in mitochondrial membrane potential and an increase of mitochondrial ROS production by functional ETC [57]. Accumulation of damaged mitochondria with swelling and cristae disruption in airway epithelial cells from COPD patients has been demonstrated [21, 22]. It has been recognized that impaired mitochondrial structural and functional integrity accompanied by excessive mtROS production is associated with enhanced programmed cell death (PCD) and cellular senescence during COPD pathogenesis, especially in the setting of altered mitophagic degradation [14, 19, 21, 22, 58] (Table 1).

**Table 1:** PINK1-PARK2-mediated mitophagy in COPD and IPF

		References
COPD		
The expression of PINK1 and PARK2		
PINK1 levels in homogenated lung	increased	[58]
PARK2 levels in homogenated lung	decreased	[19]
The role of mitophagy (the choice by the degree of damage.)		
Increased PINK1-mediated mitophagy enhancing program cell death		[58]
Insufficient PARK2-mediated accelerating cellular senescence		[19]
IPF		
The expression of PINK1 and PARK2		
PINK1 levels in AEC II	decreased	[23]
PINK1 levels in FF	decreased	[71]
PARK2 levels in FF	decreased	[40]
PARK2 levels in fibroblast	decreased	[40]
The role of mitophagy		
Insufficient PINK1-mediated mitophagy enhancing apoptosis and cellular senescence		[23, 70]
Insufficient PARK2-mediated mitophagy accelerating myofibroblast differentiation		

AEC II: type II alveolar epithelial cells

FF: fibroblast foci

PCD has been widely implicated in COPD pathogenesis, and the involvement of autophagy, including mitophagy, has been reported in not only apoptosis but also programmed necrosis, necroptosis. Although necrosis has been recognized as a mode of non-programmed cell death caused by excessive physical or chemical stress, recent advances have showed the existence of a genetically programmed form of necrosis, termed necroptosis [59]. The receptor-interacting protein-1 and -3 (RIPK1/3) kinases, which form a multiprotein complex of necrosome, are key regulators during necroptosis progression [59, 60]. In contrast to apoptosis, necroptosis can trigger both the innate and adaptive immune response through the release of highly immunologic intracellular proteins of DAMPs [61]. It has been reported that autophagy in lung tissue obtained from COPD patients is augmented due to an increased LC3B-II/LC3B-I ratio and that the Egr-1-induced LC3B expression is essential for autophagy activation [62]. LC3B−/− mouse experiments have confirmed the pivotal role of LC3B in the induction of epithelial cell apoptosis by CS exposure [63]. In terms of PINK1-mediated mitophagy, increased PINK1 protein levels with enhanced mitophagy have been implicated in COPD development. Increased PINK1-mediated mitophagy is responsible for conducting necroptosis. PINK1-deficient mice showed protection against mitochondrial dysfunction and airspace enlargement of COPD phenotypic alteration during CS exposure [58], suggesting mitophagy activation can be a detrimental process through enhancing PCD especially in the setting of cytotoxic condition.

COPD is assumed to be a disease of accelerated lung aging, and cellular senescence has been implicated in the pathogenesis of COPD, presumably due to impaired cell repopulation and the aberrant cytokine secretion observed in SASP [9, 10, 12, 14]. Autophagy plays a pivotal regulatory role in cellular senescence. CSE transiently induces the activation of autophagy followed by the accumulation of p62 and ubiquitinated proteins accompanied by an increase in HBEC senescence. The autophagy inhibition further enhances HBEC senescence with the concomitant accumulation of p62 and ubiquitinated proteins, reflecting insufficient autophagic degradation. The increased accumulation of p62 and ubiquitinated proteins detected in lung homogenates from COPD patients supports the notion that insufficient autophagic clearance is involved in the accelerated cell senescence observed in COPD [11]. To further investigate the details of the role of insufficient autophagy in the regulation of HBEC senescence, we next focused on mitophagy. Both PINK1 and PARK2 knockdown resulted in the enhancement of HBEC senescence in response to CSE exposure with concomitantly accumulated damaged mitochondria and increased ROS production [19]. Furthermore, PARK2 levels were decreased in lung homogenates from COPD patients, and there was a positive correlation between PARK2 levels and percentage of FEV1/FVC of pulmonary function test, suggesting the causal link between insufficient PARK2-mediated mitophagy and airway obstruction associated with accelerated cellular senescence during COPD pathogenesis. Although we observed potential involvement of reduced SIRT6 expression, a member of the sirtuin family, in insufficient autophagy in COPD through modulating insulin-like growth factor (IGF)-I signaling, the association between SIRT6 and PINK1-PARK2-mediated mitophagy remains to be elucidated [64].

The central purpose of both PCD and cellular senescence is eliminating damaged cells for tissue regeneration, mitophagic regulation of PCD, and cell senescence depend on the amount of damage. Hence, it is likely that once a certain threshold of mitochondrial damage is reached, the choice is made by mitophagy between PCD and senescence [19]. Accordingly, determining the adequate levels of mitophagy activation can be critically important to develop mitophagy-targeted treatment for COPD.

## PINK1-PARK2-mediated mitophagy in idiopathic pulmonary fibrosis

IPF is a progressive and devastating lung parenchymal fibrosis with poor prognosis [65]. The main pathological feature of IPF is the excessive accumulation and deposition of extracellular matrix, resulting in scar formation and loss of elasticity in lungs. Aberrant wound healing process comprised of initial alveolar epithelial cell damages of unknown cause, and subsequent accumulation of profibrotic myofibroblasts has been recognized as the key mechanisms for fibrosis development during IPF pathogenesis. Insufficient autophagy has been demonstrated in IPF lungs [66, 67], and involvement of impaired autophagy in lung fibrosis development has been clarified by increased bleomycin-induced fibrosis development in ATG4B knockout mice, an essential factor for autophagosome formation [68]. Insufficient autophagy, including mitophagy, has been implicated to IPF pathogenesis, and special attention has been paid to PINK1-PARK2-mediated mitophagy in terms of regulating cell fate for both epithelial cells and fibroblasts [20, 23, 40, 69] (Table 1). Regulatory role of PINK1-regulated mitophagy has been shown in AECIIs apoptosis and cellular senescence [23, 70]. Decrease in PINK1 expression levels accompanied by impaired autophagic degradation has been demonstrated during aging [71]. Decrease in PINK1 expression in AECIIs is elucidated in IPF lungs associated with accumulation of dysmorphic mitochondria with reduced ETC activity and increased ROS production, which leads to increased apoptosis [23]. Increased endoplasmic reticulum (ER) stress resulting from inappropriate proteostasis machinery has been detected in lung epithelial cells [72]. Intriguingly, ER stress affects mitochondrial function through downregulating PINK1, which can be attributed to expression of ATF3, a transcription repressor of PINK1 in AECIIs. Transcriptional repression of PINK1 may be associated with enhanced cellular senescence of p16 and p21 expression [23, 70]. Accordingly, reduced expression of PINK1 of insufficient mitophagy during aging and in response to ER stress in AECIIs appears to be involved in the regulatory mechanisms for cell fate with respect to IPF pathogenesis. However, the involvement of PARK2 expression levels in mitophagy regulation in AECIIs remains uncertain.

Impaired autophagy/mitophagy can also be responsible for regulating myofibroblast differentiation in lung fibroblasts [40, 67]. Both PINK1 and PARK2 reduction-mediated insufficient mitophagy can induce myofibroblast differentiation. However, compared with PINK1, PARK2 may have a predominant role in the regulation of myofibroblast differentiation. Insufficient mitophagy caused by PARK2 deficiency induces mtROS production with concomitantly activated platelet-derived growth factor receptor (PDGFR)/mammalian target of rapamycin (mTOR) signaling, resulting in myofibroblast differentiation and proliferation [40]. PARK2-deficient mice showed aggravation of bleomycin-induced lung fibrosis development. Reduced PARK2 expression levels were elucidated in myofibroblasts of fibroblastic foci and lung fibroblasts derived from IPF lungs [40]. Reduced PINK1 levels were also demonstrated in fibroblastic foci in IPF lungs, and TGF-β may have a role in PINK1 reduction, resulting in promotion and perpetuation of pulmonary fibrosis [71].

Inducing sufficient levels of PINK1-PARK2-mediated mitophagy can be a promising treatment modality to prevent lung fibrosis during IPF development. We have recently showed that pirfenidone, an anti-fibrotic agent generally used to treat IPF, induces PARK2 expression, and PARK2-mediated mitophagy is partly responsible anti-fibrotic effect of pirfenidone [73]. Intriguingly, it has been reported that thyroid hormone induces PINK1 expression with concomitant mitophagy, which is responsible for normalizing mitochondrial morphological and functional integrity and for attenuating bleomycin-induced lung fibrosis development [36].

## Conclusion

Accumulation of mitochondrial damages has an essential role in development of aging-associated pulmonary disorders. Among a wide array of mechanisms for regulating mitochondrial integrity, inappropriate PINK1-PARK2-mediated mitophagy appears to be mainly responsible for regulating cell fate, including PCD, cellular senescence, and myofibroblast differentiation during COPD and IPF pathogeneses. Therefore, modalities to achieve specific and appropriate levels of PINK1-PARK2-mediated mitophagy activation may be a promising therapeutic option to regulate the aging-associated pathology, COPD and IPF.

## Abbreviations

AEC: Airway epithelial cells
CALCOCO2 /NDP52: Calcium binding and coiled-coil domain 2
COPD: Chronic obstructive pulmonary disease
CS: Cigarette smoke
DAMPs: Damage-associated molecular patterns
DRP1: Dynamin-related protein 1
ER: Endoplasmic reticulum
ETC: Electron transport chain
FIS1: Mitochondrial fission 1 protein
FUNDC1: The fun14 domain containing 1
HBEC: Human bronchial epithelial cells
IGF: Insulin-like growth factor
IPF: Idiopathic pulmonary fibrosis
MAP1LC3/LC3: Microtubule-associated protein 1 light chain 3
MFN: Mitofusin
MFRTA: Mitochondrial free radical theory
mtDNA: Mitochondrial DNA
mTOR: Mammalian target of rapamycin
mtROS: Mitochondrial ROS
NOX4: NADPH oxidase-4
NRF: Nuclear respiration factors
OGG1: 8-Oxoguanine-DNA glycosulase-1
OPA1: Optic atrophy 1
OPTN: Optineurin
OXOHOS: Oxidative phosphorylation
PCD: Programmed cell death
PCG-1: PPARγ coactivator-1
PDGFR: Platelet-derived growth factor receptor
PFD: Pirfenidone
PINK1: PTEN-induced putative protein kinase 1
PTEN: Phosphatase and tensin homolog
RIPK3: Receptor-interacting serine/threonine-protein kinase 3
ROS: Reactive oxygen species
SASP: Senescence-associated secretory phenotype
TFAM: Mitochondrial transcription factor A
VDAC: Voltage-dependent anion channel
